# Prediction models for identifying medication overuse or medication overuse headache in migraine patients: a systematic review

**DOI:** 10.1186/s10194-024-01874-4

**Published:** 2024-10-04

**Authors:** Teerapong Aramruang, Akshita Malhotra, Pawin Numthavaj, Panu Looareesuwan, Thunyarat Anothaisintawee, Charungthai Dejthevaporn, Nat Sirirutbunkajorn, John Attia, Ammarin Thakkinstian

**Affiliations:** 1 Department of Clinical Epidemiology and Biostatistics, Faculty of Medicine, Ramathibodi Hospital, Mahidol University, Bangkok, Thailand; 2 Department of Pharmacy, Faculty of Pharmacy, Mahidol University, Bangkok, Thailand; 3 University College Cork, Cork, Ireland; 4 Department of Medicine, Faculty of Medicine, Ramathibodi Hospital, Mahidol University, Bangkok, Thailand; 5 Department of Diagnostic and Therapeutic Radiology, Division of Radiation Oncology, Faculty of Medicine, Ramathibodi Hospital, Mahidol University, Bangkok, Thailand; 6 Centre for Clinical Epidemiology and Biostatistics, School of Medicine and Public Health, University of Newcastle, Newcastle, NSW Australia

**Keywords:** Migraine, Prediction model, Machine learning, Medication overuse, Medication overuse headache, Systematic review

## Abstract

**Background:**

Migraine is a debilitating neurological disorder that presents significant management challenges, resulting in underdiagnosis and inappropriate treatments, leaving patients at risk of medication overuse (MO). MO contributes to disease progression and the development of medication overuse headache (MOH). Predicting which migraine patients are at risk of MO/MOH is crucial for effective management. Thus, this systematic review aims to review and critique available prediction models for MO/MOH in migraine patients.

**Methods:**

A systematic search was conducted using Embase, Scopus, Medline/PubMed, ACM Digital Library, and IEEE databases from inception to April 22, 2024. The risk of bias was assessed using the prediction model risk of bias assessment tool.

**Results:**

Out of 1,579 articles, six studies with nine models met the inclusion criteria. Three studies developed new prediction models, while the remaining validated existing scores. Most studies utilized cross-sectional and prospective data collection in specific headache settings and migraine types. The models included up to 53 predictors, with sample sizes from 17 to 1,419 participants. Traditional statistical models (logistic regression and least absolute shrinkage and selection operator regression) were used in two studies, while one utilized a machine learning (ML) technique (support vector machines). Receiver operating characteristic analysis was employed to validate existing scores. The area under the receiver operating characteristic (AUROC) for the ML model (0.83) outperformed the traditional statistical model (0.62) in internal validation. The AUROCs ranged from 0.84 to 0.85 for the validation of existing scores. Common predictors included age and gender; genetic data and questionnaire evaluations were also included. All studies demonstrated a high risk of bias in model construction and high concerns regarding applicability to participants.

**Conclusion:**

This review identified promising results for MO/MOH prediction models in migraine patients, although the field remains limited. Future research should incorporate important risk factors, assess discrimination and calibration, and perform external validation. Further studies with robust designs, appropriate settings, high-quality and quantity data, and rigorous methodologies are necessary to advance this field.

**Supplementary Information:**

The online version contains supplementary material available at 10.1186/s10194-024-01874-4.

## Background

Migraine is a neurological disorder characterized by severe unilateral head pain, often accompanied by nausea, vomiting, and sensitivity to light and sound [1]. It affects approximately 13.5% of the global population, and occurs more among females than males [2]. It significantly impacts on work/school productivity, family life, interictal burden, and healthcare costs [3].

Migraine is classified into episodic migraine (EM) and chronic migraine (CM) based on headache frequency [4], and treatment can be broadly categorized into acute and preventive approaches. Patients with frequent headaches and frequent use of acute medications may require preventive treatment [5, 6]. Nonetheless, migraine management remains a significant clinical challenge, resulting in insufficient medical consultation and underdiagnosis [7, 8]. Moreover, migraine treatment is often inappropriately utilized, especially preventive treatment [7]. The American Migraine Prevalence and Prevention (AMPP) study revealed that approximately 40% of migraine patients should ideally receive preventive medication but only 13.0% actually did [9]. This gap in appropriate treatment was further highlighted by the Chronic Migraine Epidemiology and Outcomes study, which found that patients with CM were less likely to receive adequate acute and preventive medications when compared to those with EM (54.2% versus 59.9%) [7]. Despite the fact that all CM patients who should receive preventive medications, only 75.6% actually did [7]; among EM patients, 24.7% met the criteria for preventive medication, but only half of these actually received it. This treatment gap leaves migraine patients at risk of medication overuse (MO).

MO occurs in approximately 15.4% of migraine patients [10], with higher in headache clinics, ranging from 34.0% to 74.3% [11, 12]. Acute MO is defined as the regular overuse of one or more drugs used for the acute treatment of migraine headaches for at least three months [1]. Treatments include specific migraine medications (i.e., ergots, triptans, or opioids on ≥ 10 days per month), and non-specific medications (i.e., non-steroidal anti-inflammatory drugs (NSAIDs), or paracetamol on ≥ 15 days per month) [1]. MO is an important factor contributing to disease progression from EM into CM [13, 14] with an odds ratio (OR) as high as 19.4 [15]. Additionally, MO plays a crucial role in the development of secondary headaches, known as medication overuse headache (MOH). MOH is characterized by an inadequate response to treatment and frequent recurrence, with 42% of patients experiencing a relapse within three years [16]. MOH also significantly impacts productivity and quality of life, and increases medical costs [17]. However, MO and MOH are used interchangeably in the context of migraine.

Addressing the challenges posed by MO/MOH necessitates the prediction of which migraine patients are at risk of developing MO/MOH. This knowledge can enable physicians to make more informed decisions regarding the prescription of preventive medications and the development of customized prevention programs tailored to at-risk migraine patients.

A prediction model is an equation constructed using various statistical methods to quantify an individual's risk of developing a specific outcome of interest [18]. Although traditional statistical modeling is widely considered as the standard technique due to its familiarity, artificial intelligence (AI) and machine learning (ML) have recently made substantial advancements in healthcare. ML offers significant advantages over traditional statistical models, such as predictor selection capabilities and handling non-parametric and non-linear interactions [19]. Consequently, risk prediction models can use traditional statistical models, AI, or ML approaches.

In the context of migraine, numerous prediction models have been introduced for various purposes, including prediction of MO/MOH [20–25]. However, to the best of our knowledge, a systematic review of prediction models for MO/MOH in migraine patients has yet to be conducted. Therefore, this systematic review aims to comprehensively summarize and criticize all available prediction models for MO/MOH in migraine patients.

## Methods

A systematic review was conducted following the preferred reporting items for systematic reviews and meta-analyses (PRISMA2020) statement [26]. Our review protocol was registered on the international prospective register of systematic reviews (PROSPERO) with registration number CRD42024532243.

### Search strategy

An extensive literature search was performed on electronic databases (i.e., Embase, Scopus, Medline via PubMed, ACM Digital Library, and IEEE Xplore) covering the period from inception to April 22, 2024 without language restrictions. The search terms were constructed based on the PICO framework, focusing on prediction models (e.g., risk score, predictive model, clinical decision model, AI, and ML) of MO/MOH in migraine patients. Detailed search strategies and specific queries are provided in Appendix 1. Identified studies were imported into EndNote software for further management.

### Eligibility criteria

All identified articles from different sources were combined, and duplicates were removed automatically by software and manually. Three reviewers (TA, NS, and AM) independently screened the titles and abstracts to select relevant studies; full articles were retrieved and reviewed if decisions could not make. A fourth reviewer (PN) resolved any disagreements. Predefined inclusion and exclusion criteria guided the selection process. Studies were eligible if they met the following criteria: 1) included migraine patients; 2) developed or externally validated prediction models (i.e., risk score, traditional statistical, or ML) of MO/MOH. Studies were excluded if they: 1) included mixed patients with migraine and other headache types; 2) were not original research (e.g., commentaries, letters to the editor, editorials); 3) published in a non-English language that could not be translated using Google Translate; and 4) published only abstracts without full papers.

### Data extraction

Data were independently extracted by the same three reviewers using a pre-specified extraction form; this included study characteristics, study design, study phase (development, internal validation, and external validation), data source, country/setting, migraine types, number of participants, baseline demographics, events per variable (EPV), patients per variable, outcomes of interest (i.e., MO/MOH and criteria,), methods for handling missing data, predictors and number of predictors, model types, and model performance including calibration and discrimination. Since the terms MO and MOH were used interchangeably in the original included studies without a clear distinction, we extracted the terms as they were originally used in the included studies.

### Risk of bias assessment

Risk of bias (ROB) evaluates the models to estimate their transparency, bias, and applicability. This was performed using the Prediction Model Risk of Bias Assessment Tool (PROBAST) [27, 28] considering four domains: participants, predictors, outcome, and analysis. Signaling questions are answered “yes”, “probably yes”, “probably no”, “no”, or “no information”, where “yes” indicates low risk of bias and “no” indicates high risk of bias. The overall ROB is judged low if all domains are considered low risk and high if at least one domain is considered high risk.

## Results

### Study search

**Fig. 1:**
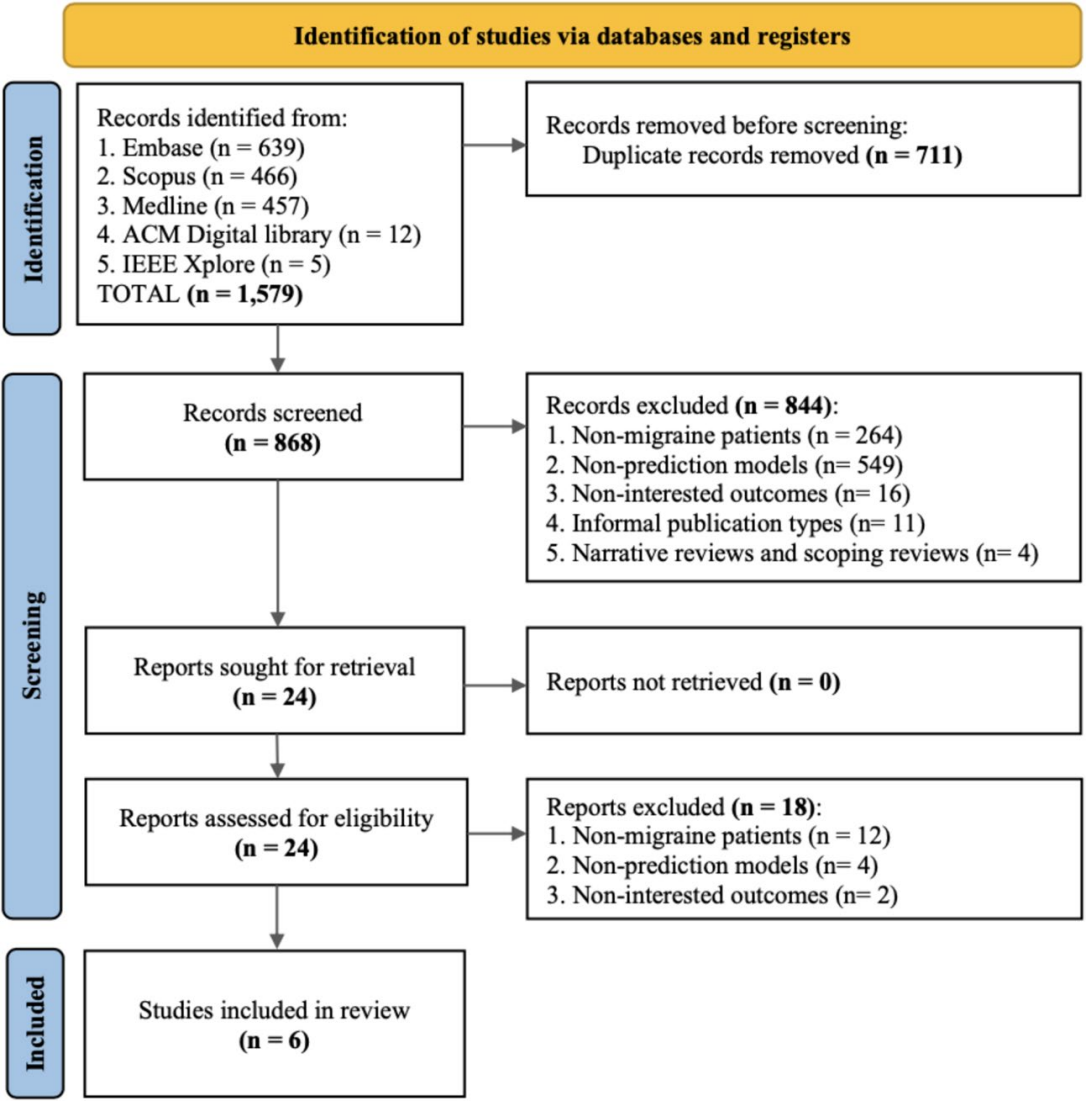
PRISMA flow diagram for study selection

A total of 1,579 records were identified. Six studies met the eligibility criteria after removing duplicates and screening titles, abstracts, and full papers. The reasons for excluding other studies are detailed in Fig. 1.

### Characteristics of included studies

Characteristics of included studies are described in Tables 1 and 2. Half of the studies (50%) focused on model development [21–23], while the remaining studies conducted validation of other scores [20, 24, 25]. Among the model development studies, one exclusively derived a model [21], whereas the others performed both model derivation and internal validation [22, 23]. Most of the studies (5, 83.3%) were cross-sectional designs [20, 21, 23–25]; only one study employed a cohort approach [22]. All studies utilized data from prospective data collection, with one study also incorporating additional data from electronic medical records (EMRs) [22]. The study settings were mainly headache clinics (4, 66.7%) [22–25].

**Table 1:** Characteristics of the studies included in this review

Author	Year	Phase	Study design	Data source	Setting	Types of migraine	No. of patients (n)	No. of MO/MOH (n, %)	Age, year (mean)	Sex, male (%)	Country
Grande RB [20]	2009	Validation of other scores*	Cross-sectional	Prospective data collection	General population	CM only	17	14, 82.4	-	-	Norway
Onaya T [21]	2013	Derivation	Cross-sectional	Prospective data collection	Neurological clinic	Any migraine	69	22, 31.9	38.0	10.1	Japan
Mose LS [22]	2018	Derivation, Internal validation	Cohort	Prospective data collection and EMRs	Headache clinic	Any migraine	131	16, 12.2	39.0	12.2	Denmark
Ferroni P [23]	2020	Derivation, Internal validation	Cross-sectional	Prospective data collection	Headache and pain clinic	EM and CM	777	162, 20.8	40.5	18.3	Italy
Wang YF [24]	2023	Validation of other scores*	Cross-sectional	Prospective data collection	Headache clinic	CM only	563	320, 56.8	41.7	19.9	Taiwan
Wang YF [25]	2023	Validation of other scores*	Cross-sectional	Prospective data collection	Headache clinic	CM only	1419	799, 56.3	41.7	20.0	Taiwan

^*^Modified previous behavioral dependency score and validated in CM patients

*Abbreviations: CM* chronic migraine, *EM* episodic migraine, *EMRs* electronic medical records, *MO* medication overuse, *MOH* medication overuse headache

**Table 2:** Characteristics of the studies included in this review

Author	Year	MO/MOH criteria	MO/MOH identification	No. of predictors (n)	EPV	Patients per variable	Outcome term	Missing data handling	Predictor selection	Internal validation
Grande RB [20]	2009	ICHD-II and relevant revisions	Neurological residents	5	2.8	3.4	MO	Complete case analysis	-	-
Onaya T [21]	2013	ICHD-II with revision	-	30	0.7	2.3	MOH	-	Univariate, Multivariate stepwise LR	-
Mose LS [22]	2018	ICHD-III beta	Neurologists	20	0.8	6.6	MOH	Complete case analysis	Not perform	Bootstrap resampling
Ferroni P [23]	2020	-	Neurologists	53	3.1	14.7	MO	PVI	Not perform	Hold-out testing set
Wang YF [24]	2023	ICHD-III	Headache specialists	10	32.0	56.3	MOH	-	-	-
Wang YF [25]	2023	ICHD-III	Headache specialists	5, 10	159.8, 79.9	283.8, 141.9	MOH	Complete case analysis	-	-

*Abbreviations: CV* cross validation, *EPV* events per variable, *ICHD* The International Classification of Headache Disorders, *LR* logistic regession, *MO* medication overuse, *MOH* medication overuse headache, *PVI* predictive value imputation

The number of participants in the development phase ranged from 69 to 777; for the validation phase it ranged from 17 to 1,419. The types of migraine included CM only (*N* = 3) [20, 24, 25], any migraine (*N* = 2) [21, 22], and both EM and CM (*N* = 1) [23].

Most studies (4, 66.7%) used the term MOH as the outcome of interest [21, 22, 24, 25]. The criteria for identifying MO/MOH followed the International Classification of Headache Disorders (ICHD) versions II (*N* = 2) [20, 21], III (*N* = 2) [24, 25], and III beta (*N* = 1) [22], with one study not reporting the criteria used [23]. Detailed information on the terms used for outcomes, along with their definitions and the guidelines applied in the included studies, can be found in Appendix 2.

### Included predictors in prediction models

The predictors considered in the included studies can be categorized into several domains: patient demographics (12 predictors), family history (1 predictor), physical examinations (12 predictors), migraine and related characteristics (13 predictors), underlying diseases and symptoms (6 predictors), laboratory tests (26 predictors), genetic data (13 predictors), medications (4 predictors), and questionnaire-based evaluations (4 predictors). Detailed descriptions of each model's predictors and data types are provided in Table 3 and Appendix 3. The predictors per study ranged from 5 to 53, with a median of 10 (IQR: 8–25). The most commonly used predictors were gender (*N* = 3) [21–23] and age (*N* = 3) [21–23]. Genetic data were included in 2 studies (33.3%) [21, 23] and questionnaire-based variables were utilized in 5 studies (83.3%). The questionnaires included the assessment of migraine disability (Migraine Disability Assessment Score (MIDAS) [29, 30]), dependence behaviors (Severity of Dependence Scale (SDS) [31], Leeds Dependence Questionnaire (LDQ) [32]), and personality traits (NEO Five-Factor Inventory (NEO-FFI) [33]). The EPV ranged from 0.7 to 3.1 for model development, and from 2.8 to 158.8 in the validation phase.

**Table 3:** Predictors considered in prediction model in each study

Predictors considered in model	Study					
	Grande RB, 2009 [20]	Onaya T, 2013 [21]	Mose LS, 2018 [22]	Ferroni P, 2020 [23]	Wang YF, 2023 [24]	Wang YF, 2023 [25]
**Patient demographics**						
Gender	-	C	C	C	-	-
Age	-	S	S	S	-	-
Marital status	-	-	C	-	-	-
Education	-	-	C, S	-	-	-
Occupation	-	-	C	-	-	-
Physical activity	-	-	S	-	-	-
Sleep habit	-	-	S	-	-	-
Alcohol and coffee intake	-	-	-	C	-	-
Smoking	-	-	-	C	-	-
Dietary intake	-	C	-	-	-	-
Menopausal status	-	-	-	C	-	-
Age at menarche	-	-	-	S	-	-
**Family history**						
Migraine in family	-	C	-	C	-	-
**Physical examinations**						
BP	-	-	-	S	-	-
BMI	-	-	-	S	-	-
**Migraine and related characteristics**						
Type of migraine	-	C	-	C	-	-
Age of onset migraine	-	C	-	S	-	-
Length of chronicization	-	-	-	S	-	-
Headache frequency	-	-	-	S	-	-
Pain localization	-	C	-	C	-	-
Characteristics of pain	-	C	-	-	-	-
Unilateral cranial autonomic symptoms	-	-	-	C	-	-
Dopaminergic symptoms	-	-	-	C	-	-
Concomittant with CH	-	-	-	C	-	-
Concomittant with TTH	-	-	C	C	-	-
Relation with menstruation	-	C	-	-	-	-
Relation with stress and uneasiness	-	C	-	-	-	-
No. of consultation with headache clinic	-	-	S	-	-	-
**Underlying diseases and symtoms**						
Depression	-	C	-	-	-	-
Neuropsychiatric	-	-	-	C	-	-
Cardiovascular	-	-	-	C	-	-
Endocrine-metabolic	-	-	-	C	-	-
Motion sickness	-	C	-	-	-	-
**Laboratories**						
CBC panel	-	-	-	S	-	-
Chem panel	-	-	-	S	-	-
Lipid panel	-	-	-	S	-	-
Renal panel	-	-	-	S	-	-
LFT panel	-	-	-	S	-	-
**Genetics**						
Genetic polymorphisms*	-	C	-	C	-	-
**Medications**						
Class of acute medication (NSAIDs, triptans, or others)	-	-	-	C	-	-
Response to triptans	-	-	-	C	-	-
Use of at least one preventive medication	-	-	-	C	-	-
COCs	-	-	-	C	-	-
**Questionnaire-based evaluations**						
MIDAS	-	-	S	-	-	-
Modified SDS	S	-	-	-	-	S
Modified LDQ	-	-	-	-	S	S
NEO-FFI	-	S	S	-	-	-

^*^See Appendix 3 for more details of associated polymorphisms

*Abbreviations: BMI* body mass index, *BP* blood pressure, *CBC* complete blood count, *CH* cluster headache, *Chem* blood chemistry, *COCs* combined oral contraceptives, *LDQ* Leeds Dependence Questionnaire, *LFT* liver function test, *MIDAS* Migraine Disability Assessment Score, *NEO-FFI* NEO Five-Factor Inventory questionnaire, *SDS* Severity of Dependence Scale, *TTH* tension-type headache, *C* used in categorized form, *S* used in scaled form

### Model development and performance

A total of 9 analytical models were evaluated across 6 eligible studies, with 2 studies utilizing more than one model [23, 25]. The model types and model performance of the included studies stratified by study phases are provided in Table 4.

**Table 4:** Analytical method and performance of the included studies stratified by study phases

Author	Year	Analytical method	Discrimination					
			**Derived model**		**Internal validation**		Validation of other scores*	
			AUROC (95% CI)	Other matrices	AUROC (95% CI)	Other matrices	AUROC (95% CI)	Other matrices
Grande RB [20]	2009	ROC analysis of modified SDS	-	-	-	-	-	Mean SDS scores: CM, 4.5; CM + MO/MOH, 6.2
Onaya T [21]	2013	Logistic regression	-	Mean PI score (SD): Migraine, 4.62 ± 1.83; MO/MOH, 7.32 ± 1.60 (P < 0.001)	-	-	-	-
Mose LS [22]	2018	LASSO regression	-	-	0.62 (0.41–0.82)	-	-	-
Ferroni P [23]	2020	SVM baseline	0.71 (0.67–0.75)	Sen: 0.44, Pre: 0.86, F1: 0.58	0.81 (0.76–0.86)	Sen: 0.63, Pre: 0.96, F1: 0.76	-	-
		SVM-RO	0.79 (0.75–0.82)	Sen: 0.79, Pre: 0.47, F1: 0.59	0.81 (0.75–0.86)	Sen: 0.74, Pre: 0.33, F1: 0.45	-	-
		SVM-RO with combination	0.77 (0.73–0.80)	-	0.83 (0.78–0.88)	Sen: 0.69, Spec: 0.87, Acc: 0.87	-	-
Wang YF [24]	2023	ROC analysis of modified LDQ	-	-	-	-	0.85 (0.82–0.88)	Sen: 0.78, Spec: 0.77
Wang YF [25]	2023	ROC analysis of modified SDS	-	-	-	-	0.84	Sen: 0.73, Spec: 0.80
		ROC analysis of modified LDQ	-	-	-	-	0.85	Sen: 0.76, Spec: 0.78

^*^Modified previous behavioral dependency score and validated in CM patients

*Abbreviations: Acc* accuracy, *AUROC* area under the receiver operating characteristic curve, *CI* confidential interval, *CM* chronic migraine, *F1* f1-score, *LASSO* least absolute shrinkage and selection operator, *LDQ* Leeds Dependence Questionnaire, *MO* medication overuse, *MOH* medication overuse headache, *PI* predictive index, *Pre* precision, *RO* random optimization, *ROC* Receiver Operating Characteristics, *SD* standard deviation, *SDS* Severity of Dependence Scale, *Sen* sensitivity, *Spec* specificity, *SVM* support vector machines

Two studies employed traditional statistical models [21, 22], and one used ML [23] for model development. The models included logistic regression [21], least absolute shrinkage and selection operator (LASSO) regression [22], and support vector machines (SVM) [23]. The discrimination performance of the models, measured by the area under the receiver operating characteristic (AUROC) curve, ranged from 0.71 to 0.79 for the derivation phase and from 0.62 to 0.83 for internal validation. One study did not report model performance but noted that the risk score for migraine with MO/MOH was statistically higher than for migraine patients (*P* < 0.001) [21].

For the validation of other scores, all studies employed questionnaire-based tools to detect MO/MOH, specifically utilizing the modified SDS [20, 25] and the modified LDQ [24, 25]. The optimal cutoff scores for the SDS and LDQ in identifying MO/MOH were determined through receiver operating characteristic (ROC) curve analysis across all studies. The AUROC for the validation of these scores ranged from 0.84 to 0.85. One study did not report model performance but observed a higher risk score for CM patients with MO/MOH compared to those without MO/MOH [20].

### Risk of *bias* assessment

**Fig. 2:**
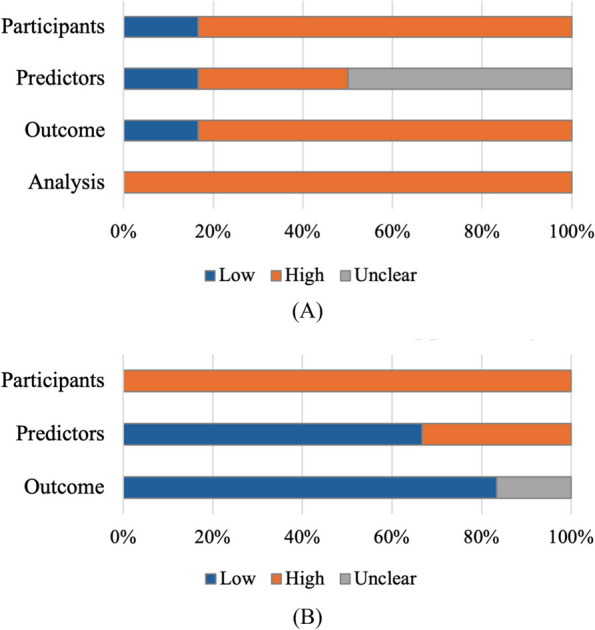
Risk of bias (**A**) and applicability (**B**) assessment using the PROBAST based on four domains of prediction models for outcome prediction in MO/MOH

The results of the PROBAST assessment are detailed in Table 5 and Fig. 2. Overall, all studies were assessed as having high ROB and concerns regarding applicability. Five studies were rated as high ROB in the participants domain due to their cross-sectional design [20, 21, 23–25]. For applicability, all studies were rated as high concern due to studies focusing on specific types of migraine [20, 24, 25] and including participants from specific settings [20–25]. In the predictors domain, two studies were rated as high ROB because they included genetic data as predictors [21, 23], which might not be practical at the time of intended use. Clarity about whether predictor assessments were made without knowledge of the outcome was lacking, leading to three studies being rated as unclear ROB in this domain [20, 24, 25]. For applicability, genetic data raised concerns in some hospital settings, resulting in two studies being rated as high concern [21, 23]. Five studies were rated as high ROB in the outcomes domain, primarily because predictors and outcomes were assessed at the same point and lack of clarity about whether outcomes were determined without knowledge of predictor information [20, 21, 23–25]. The overall applicability in the outcome domain was rated as low ROB, except for one study where the definition of MO/MOH was unclear [23]. For the analysis domain, all studies were rated as high ROB, mainly due to the insufficient number of participants, as indicated by EPV of less than 10 for model development [21–23] and less than 100 for validation of other scores [20, 24, 25], as well as the lack of calibration [20–25] based on PROBAST assessment [27, 28].

**Table 5:** Tabular presentation for the PROBAST assessment of included studies

Study	ROB				Applicability			Overall	
	Participants	Predictors	Outcome	Analysis	Participants	Predictors	Outcome	ROB	Applicability
Grande RB, 2009 [20]	−	?	−	−	−	+	+	−	−
Onaya T, 2013 [21]	−	−	−	−	−	−	+	−	−
Mose LS, 2018 [22]	+	+	+	−	−	+	+	−	−
Ferroni P, 2020 [23]	−	−	−	−	−	−	?	−	−
Wang YF, 2023 [24]	−	?	−	−	−	+	+	−	−
Wang YF, 2023 [25]	−	?	−	−	−	+	+	−	−

*Abbreviations:* *ROB* risk of bias, + indicates low ROB/low concern regarding applicability, − indicates high ROB/high concern regarding applicability, ? indicates unclear ROB/unclear concern regarding applicability

## Discussion

We conducted a systematic review of prediction models for identifying MO/MOH in migraine patients. Six studies with nine eligible prediction models were included in this review. Our findings indicate that the number of studies focused on MO/MOH prediction models remains limited, with considerable variation in the models and datasets utilized across the studies. As a result, a meta-analysis to pool model performance could not be performed. Nonetheless, the overall results are promising. Two studies developed models based on traditional statistical approaches [21, 22], one used a ML approach [23], and the remaining three employed ROC analysis of modified scores [20, 24, 25]. Most models yielded relatively acceptable discriminative performance in derivation, internal validation, and validation of other scores. However, none of the included studies conducted external validation to verify generalizability or carried out prospective evaluations in real clinical settings, highlighting a significant gap in the research. In addition, these models were subject to a high risk of bias.

Furthermore, the initial study that validated the SDS score in CM patients primarily aimed to validate its use in primary chronic headache. However, a subgroup analysis focusing on CM patients was conducted. Despite this, the study did not report discriminative performance metrics but indicated that CM patients with MO/MOH had higher scores than those without MO/MOH [20]. Validation of the modified LDQ score in CM patients was addressed in two studies by the same author. The first paper was published in January 2023 [24], followed by a second paper in November of the same year [25]. These studies had sample sizes of 563 and 1419, respectively. Given the potential similarity in protocols and the proximity of publication dates, there is a possibility of participant overlap between the two studies.

The terminology for interested outcomes in the original included studies were varied, with two studies using the term MO [20, 23] and others using MOH [21, 22, 24, 25]. The distinction between MO and MOH is not clearly articulated, as both terms exist in a gray area where a precise differentiation is often not possible. Within the context of migraine, MO and MOH are used interchangeably. Appendix 2 provides detailed definitions and the guidelines used to define them in the original included studies. Highlighting this interchangeability, the modified SDS score was validated for predicting both MO [20] and MOH [25] as outcomes, underscoring the interchangeable nature of these two entities. Furthermore, variations exist in the criteria used to identify MO/MOH. One study relied on the ICHD-II criteria [20, 21], while three employed the ICHD-III criteria [22, 24, 25], resulting in different MO/MOH detection rates. Notably, when transitioning from ICHD-II to ICHD-III criteria, the frequency of MO/MOH detection increased fourfold [34]. This discrepancy necessitates careful consideration.

Comparing model performances among studies presents a challenge due to the diverse participant characteristics, predictors, data sources, and data collections in the datasets. Our review indicated promising results regarding ML model performance, with an AUROC of 0.83 [23], surpassing the traditional statistical model, LASSO regression (AUROC of 0.62) [22] for interval validation. Notably, only one study employed the ML approach using SVM [23], a method capable of identifying the optimal hyperplane for effectively separating data points into distinct target classes [35]. Consequently, there is substantial potential for improvement in model development for MO/MOH prediction in migraine. Analyzing discrimination performance through AUROC provides insights into the model's sensitivity and specificity. In this context, a false positive is favored over a false negative. A false positive occurs when the model predicts patients who do not have MO/MOH as having these conditions, whereas a false negative occurs when patients do have MO/MOH but are classified by the model as not having these conditions. When comparing models between studies, it is necessary to consider this point. Sensitivity and specificity are point estimates, and the trade-off between these metrics depends on the cut-off value. Therefore, the initial assessment should focus on AUROC, as it serves as a reliable indicator of the model’s overall discrimination power. Nevertheless, this review reveals that most studies report model performance based on AUROC, neglecting the insights provided by other metrics, such as the area under the precision-recall curve and the F1-score. These metrics are particularly useful for imbalanced datasets, which reflect real-world settings where only a subset of migraine patients experience MO/MOH.

AI and ML have been introduced into various applications, including healthcare. Concerns regarding the black-box nature of ML approaches need to be addressed, particularly in the healthcare context [36]. Physicians and patients require explainable models to inform decision-making in a clinical setting. For instance, in the context of migraine treatment, such explainable models could assist physicians in deciding whether to prescribe preventive migraine treatments to patients at risk of MO/MOH. This review found that the best-performing model in internal validation was developed using the ML approach, which is often difficult to explain [23]. In contrast, the traditional statistical approach, specifically regression analysis, is considered more transparent but generally exhibits poorer performance in this situation [22]. Further research should better explain AI and ML approaches and improve model transparency. The ML-based study included in this review employed random optimization (RO) to interpret the importance of predictor groups within the model [23]. However, useful methods such as Local Interpretable Model-agnostic Explanations (LIME) [37] and SHapley Addictive exPlanations (SHAP) [38] could be beneficial and should be further utilized to make models more explainable in the future research.

The study design and setting are crucial for developing prediction models, particularly MO/MOH, in the context of migraine. In this review, the studies included various types of migraine patients and a range of settings. To address this issue, we recommend that further research perform external validation. We suggest including all types of migraine patients and clinical settings to reflect real-world clinical scenarios better. Furthermore, most studies utilized cross-sectional data [20, 21, 23–25], which limits distinguishing between cause and effect and does not address time-varying covariates, an advantage of longitudinal data. For predicting MO/MOH in migraine, an appropriate design would involve using cohort data where participants initially do not exhibit the outcome, and the outcome is then assessed at a subsequent time point relative to the predictive factors [39]. In addition, in the context of migraine, patients are often followed up over multiple visits, during which they may be diagnosed with new comorbidities or prescribed new medications. As many important risk factors vary over time, considering these temporal changes within dynamic models would provide a more accurate reflection of real-world data. Specifically, employing longitudinal data modeling, such as Cox proportional hazards model [40], random survival forests [41], and gradient boosting [42], could be particularly beneficial. These approaches would align more closely with real clinical settings and improve model performance.

Most included studies had small sample sizes, with participant numbers ranging from 69 to 777 during the development phase and 17 to 1,419 for validation of other scores. In model development based on limited sample size, there is an increased likelihood of selecting unimportant predictors while omitting important predictors from the model, potentially leading to overfitting [43]. According to PROBAST recommendations [27, 28], EPV should ideally be at least 10, increasing to 20 or more for each predictor during model development. For external validation, the aim is an EPV of 100. In our review, the number of predictors integrated into the models ranged from 20 to 53 for model development and 5 to 10 for validation of other scores. Therefore, to establish robust models for MO/MOH, it is estimated that between 200 to 530 participants with MO/MOH are required for model development and 500 to 1,000 for validation of other scores or external validation. Notably, apart from one study validating the modified SDS in CM patients [25], none of the included studies met these criteria, potentially leading to an over-optimistic evaluation of the model's performance.

Several strategies can be employed to address the issue of limited participant numbers. One potential solution is to encourage data sharing to accumulate more information independently. However, careful consideration must be given to the methodologies used to collect and assess predictors across different datasets. Utilizing secondary data (e.g., EMRs or real-world data) presents an opportunity to derive further benefits, providing detailed patient care information in both structured (e.g., diagnosis codes) and unstructured formats (e.g., clinical notes and images). To utilize such data effectively, rigorous steps, including data standardization and harmonization, cohort construction, variable and outcome curation, and robust modeling techniques, are essential [44]. However, in our review, only one study leveraged EMRs data solely to validate outcomes rather than maximize the advantages offered by real-world data sources [22]. In dealing with missing data, aside from typical approaches like complete case analysis or imputation with predictor averages from the training set used in this review, more valid and comprehensive methods such as Multivariate Imputation by Chained Equations (MICE) [45] can be employed. MICE aims to retain the relationships between predictors in the original dataset while reducing bias introduced by imputed values, but it can also be computationally expensive, especially for large datasets.

There were variations in the risk factors considered in prediction models across different studies, and certain risk factors associated with MO/MOH in migraines were completely omitted from these prediction models [10, 46, 47]. For example, risk factors related to underlying disease/symptom and medication, including substance-related disorders (OR, 7.60), insomnia (OR, 5.59), traumatic head injury (OR, 3.54), snoring (OR, 2.24), anxiety (OR, 2.61), cutaneous allodynia (OR, 1.22), and the previous and current use of combined oral contraceptives (COCs) (OR, 3.38). While some risk factors associated with MO/MOH were omitted from the existing models, these models demonstrated promising discriminative performance. This finding may be attributed to the inclusion of predictors with strong pathophysiological relevance. There are relations between modified SDS/LDQ scores and substance-related disorders, sleep habits and insomnia, as well as depression/neuropsychiatric diseases and anxiety. For example, there are established links between modified SDS/LDQ scores and substance-related disorders. One study indicated that approximately 70 percent of MO/MOH patients met the diagnostic criteria for substance-related disorders [48]. The pathophysiological mechanisms underlying this dependency involve processes such as central sensitization and structural plasticity within dopamine regulation pathways [48]. The SDS and LDQ are specifically designed to measure dependence behaviors, which, alongside substance-related disorders, provide valuable insights into the dependence behaviors observed in MO/MOH patients. An important predictor for MO/MOH is the frequency of acute medication use, which is also a component of the diagnostic criteria [1]. However, this predictor should not be included as a predictor in prediction model due to concerns raised by the PROBAST guidelines. According to PROBAST, this predictor is unavailable at the point of model application and integral to outcome determination, resulting in a high ROB and concerns about applicability in clinical settings [27, 28].

Moreover, none of the studies integrated data concerning patient care aspects, such as the timing of initiating preventive medication, the type of preventive medication, or variables related to physicians like medical specialists or years of experience. Incorporating these additional variables could potentially enhance the predictive performance of the models. When aiming to implement these models in clinical settings to serve as clinical decision support, the variables considered must be easily measurable, routinely available in everyday medical practice, or have information that patients already know. Variables requiring specialized techniques for measurement (e.g., genetic testing, biomarkers) may be impractical for widespread application in the real world, particularly in areas with limited resources. However, in settings where genetic screening is routine, models integrating genetic information alongside other risk factors [21, 23] demonstrated superior performance compared to models without genetic data [22]. Conversely, if the ultimate objective is to develop an automatic risk prediction model for deployment within hospital information systems, variables reliant on manually obtained questionnaire data may not be suitable for this situation [20–22, 24, 25].

Although these prediction models showed acceptable performance in the discrimination of MO/MOH in migraine patients, they were at high ROBs for many reasons. The majority were cross-sectional design [20, 21, 23–25], predictors and outcomes were assessed at the same points [20, 21, 23–25], there was lack of calibration [20–25], and low EPV for the construct of the model [21–23] and validation of other scores [20, 24, 25]. Regarding applicability, there were high concerns about the generalizability of the models due to specific types of migraine [20, 23–25], and specific settings [20–25].

The development of a reliable prediction model for MO/MOH in migraine patients would yield significant benefits from both healthcare provider and patient perspectives. Such a model would enable early detection of patients at risk for MO/MOH, facilitating timely intervention before the conditions occured. Early identification could assist physicians in prescribing preventive migraine medications [49], which are advantageous in reducing the escalation of acute medication use and thereby preventing MO/MOH [50, 51]. The early initiation of preventive treatments results in a reduction in migraine days, symptom intensity, attack duration, improved response to acute treatments, and prevention of disease progression [50, 51]. Furthermore, patient education is crucial for effective migraine management. Once patients are aware of their risk for MO/MOH, healthcare providers can educate them about this condition, teach them to monitor their headache and acute medication use, and encourage maintaining a headache diary [52, 53]. Physicians might also closely monitor patients at risk [53]. Additionally, physicians can promote lifestyle modifications to address modifiable risk factors, such as cessation of tobacco use and increased physical activity [10, 46, 47]. These comprehensive approaches can significantly enhance the management and outcomes for migraine patients at risk of MO/MOH.

To the best of our knowledge, this study presents the first and only systematic review that comprehensively evaluates all available prediction models for identifying MO/MOH in migraine patients. The review examined various aspects, including study characteristics, predictors utilized, model types and analytical methods, and model performance. The findings from our study provide valuable insights into the development of prediction models for MO/MOH in migraine patients. Nevertheless, this systematic review has limitations. Given the variations in reporting model performance metrics, we were not able to formally pool algorithms in a summary model.

## Conclusion

This systematic review comprehensively evaluates existing prediction models for identifying MO/MOH in migraine patients. An ongoing need remains to develop reliable prediction models, potentially using ML approaches. Significant risk factors associated with MO/MOH and variables related to patient care and physician characteristics should be incorporated to enhance predictive performance and clinical relevance. Evaluating models beyond the AUROC and including calibration are crucial for improving model performance. Additionally, the need for studies performing external validation is noteworthy. Emphasizing the importance of critical considerations for developing accurate prediction models, including study design, setting, data quality and quantity, and research methodologies, will ensure practical application in real-world clinical settings.

## Supplementary Information

Supplementary Material 1.

Supplementary Material 2.

Supplementary Material 3.

## Data Availability

No datasets were generated or analysed during the current study.

## Abbreviations

AI: Artificial intelligence
AUROC: Area under the receiver operating characteristic
CM: Chronic migraine
EM: Episodic migraine
EMRs: Electronic medical records
EPV: Events per variable
ICHD: The International Classification of Headache Disorders
LASSO: Least absolute shrinkage and selection operator
LDQ: Leeds Dependence Questionnaire
ML: Machine learning
MO: Medication overuse
MOH: Medication overuse headache
OR: Odds ratio
PROBAST: The Prediction Model Risk of Bias Assessment Tool
ROB: Risk of bias
ROC: Receiver operating characteristic
SDS: Severity of Dependence Scale
SVM: Support vector machines

## Availability of data and materials

Data sharing is not applicable to this article as no datasets were generated or analysed during the current study.
