# Supplementary material for: Prediction models for identifying medication overuse or medication overuse headache in migraine patients: a systematic review
Source: J Headache Pain. 2024 Oct 4;25(1):165. doi: 10.1186/s10194-024-01874-4 (PMC11450990; doi:10.1186/s10194-024-01874-4)
Supplement: Supplementary file 1 — Supplementary Material 1. [file 10194_2024_1874_MOESM1_ESM.pdf]

Appendix 1 Search terms and results

Database: Embase (last searching on April 22, 2024)

Search Code	Terms	Results
**Populations (P)**		
#1	'migraine'/exp	92,136
#2	migraine	92,121
#3	'migraine headache'	4883
**#4**	**#1 OR #2 OR #3**	**92,136**
**Intervention and Comparator (IC)**		
#5	'risk score'/exp	71
#6	'risk prediction model'/exp	85
#7	'risk model'	13,995
#8	questionnaire	1,275,375
#9	#5 OR #6 OR #7 OR #8	1,289,079
#10	'predictive model'/exp	15,684
#11	'prediction model'/exp	217
#12	predicting	410,153
#13	'prediction tool'	4,392
#14	'prediction rule'	2,511
#15	#10 OR #11 OR #12 OR #13 OR #14	426,571
#16	'clinical decision support system'/exp	6,835
#17	'clinical decision rule'/exp	874
#18	'clinical decision model'	62
#19	'clinical prediction score'	218
#20	#16 OR #17 OR #18 OR #19	7,964
#21	'artificial intelligence'/exp	101,305
#22	'machine learning'/exp	468,406
#23	#21 OR #22	506,109
**#24**	**#9 OR #15 OR #20 OR #23**	**2,151,850**
**Outcome (O)**		
#25	'medication overuse'/exp	1,197
#26	'drug induced headache'/exp	19,918
#27	'medication overuse headache'	2,043
#28	'medication over*'	5,439
#29	'medication misuse'	418
#30	'MOH'	14,217
#31	'analgesic rebound headache'	32
**#32**	**#25 OR #26 OR #27 OR #28 OR #29 OR #30 OR #31**	**37,111**
**Combine P, IC, and O**		
**#33**	**#4 AND #24 AND #32**	**639**

Database: Scopus (last searching on April 22, 2024)

Search Code	Terms	Results
**Populations (P)**		
#1	migraine	76,315
#2	"migraine headache"	5,881
**#3**	**#1 OR #2**	**76,315**
**Intervention and Comparator (IC)**		
#4	"risk score"	51,256
#5	"risk prediction"	25,464
#6	"risk model"	24,299
#7	questionnaire	1,669,999
#8	#4 OR #5 OR #6 OR #7	723,835
#9	predicting	409,695
#10	"prediction tool"	10,964
#11	"prediction rule"	3,464
#12	"predictive model"	95,419
#13	"prediction model"	154,866
#14	#9 OR #10 OR #11 OR #12 OR #13	926,453
#15	"clinical decision model"	95
#16	"clinical decision rule"	2,529
#17	"clinical prediction score"	213
#18	"decision support systems"	129,855
#19	#15 OR #16 OR #17 OR #18	132,325
#20	"artificial intelligence"	554,334
#21	"machine learning"	610,547
#22	#20 OR #21	1,061,216
**#23**	**#8 OR #14 OR #19 OR #22**	**3,590,159**
**Outcome (O)**		
#24	"medication overuse"	2,790
#25	"medication overuse headache"	1,387
#26	"medication over*"	4,567
#27	"medication misuse"	381
#28	"MOH"	4,174
#29	"drug-induced headache"	18,903
#30	"analgesic rebound headache"	42
**#31**	**#24 OR #25 OR #26 OR #27 OR #28 OR #29 OR #30**	**26,484**
**Combine P, IC, and O**		
**#32**	**#3 AND #23 AND #31**	**466**

Database: Medline/PubMed (last searching on April 22, 2024)

Search Code	Terms	Results
**Populations (P)**		
#1	"Migraine Disorders"[Mesh]	32,755
#2	migraine	48,961
#3	“migraine headache”	3,088
**#4**	**#1 OR #2 OR #3**	**48,961**
**Intervention and Comparator (IC)**		
#5	“risk score”	31,337
#6	“risk prediction”	18,701
#7	“risk model”	9,167
#8	questionnaire	1,614,794
#9	#5 OR #6 OR #7 OR #8	1,663,441
#10	predicting	1,978,486
#11	“prediction tool”	2,927
#12	“prediction rule”	1,785
#13	“predictive model”	18,848
#14	“prediction model”	29,287
#15	#10 OR #11 OR #12 OR #13 OR #14	1,978,486
#16	"Decision Support Systems, Clinical"[Mesh]	9,729
#17	"Clinical Decision Rules"[Mesh]	952
#18	“clinical decision model”	46
#19	“clinical prediction score”	132
#20	#16 OR #17 OR #18 OR #19	10,840
#21	"Artificial Intelligence"[Mesh]	193,703
#22	"Machine Learning"[Mesh]	66,665
#23	#21 OR #22	193,703
**#24**	**#9 OR #15 OR #20 OR #23**	**3,577,522**
**Outcome (O)**		
#25	"Headache Disorders, Secondary"[Mesh]	3,546
#26	“medication overuse”	1,716
#27	“medication overuse headache”	1,134
#28	“medication over*”	1,916
#29	“medication misuse”	297
#30	“MOH”	7,193
#31	“drug-induced headache”	67
#32	“analgesic rebound headache”	23
**#33**	**#25 OR #26 OR #27 OR #28 OR #29 OR #30 OR #31 OR #32**	**11,884**
**Combine P, IC, and O**		
**#34**	**#4 AND #24 AND #33**	**457**

Database: ACM Digital library (last searching on April 22, 2024)

Search Code	Terms	Results
**Populations (P)**		
#1	migraine	456
#2	“migraine headache”	18
**#3**	**#1 OR #2**	**456**
**Intervention and Comparator (IC)**		
#4	“risk score”	696
#5	“risk prediction”	840
#6	“risk model”	546
#7	questionnaire	47,444
#8	#4 OR #5 OR #6 OR #7	48,988
#9	predicting	217,680
#10	“prediction tool”	505
#11	“prediction rule”	310
#12	“predictive model”	5,780
#13	“prediction model”	11,884
#14	#9 OR #10 OR #11 OR #12 OR #13	217,695
#15	“clinical decision model”	2
#16	“clinical decision rule”	2
#17	“clinical prediction score”	0
#18	“decision support systems”	10,206
#19	#15 OR #16 OR #17 OR #18	10,209
#20	“artificial intelligence”	149,207
#21	“machine learning”	132,970
#22	#20 OR #21	165,897
**#23**	**#8 OR #14 OR #19 OR #22**	**363,524**
**Outcome (O)**		
#24	“medication overuse”	6
#25	“medication overuse headache”	2
#26	“medication over*”	33
#27	“medication misuse”	3
#28	“MOH”	347
#29	“drug-induced headache”	2
#30	“analgesic rebound headache”	1
**#31**	**#24 OR #25 OR #26 OR #27 OR #28 OR #29 OR #30**	**375**
**Combine P, IC, and O**		
**#32**	**#3 AND #23 AND #31**	**12**

Database: IEEE Xplore (last searching on April 22, 2024)

Search Code	Terms	Results
**Populations (P)**		
#1	migraine	256
#2	"migraine headache"	61
**#3**	**#1 OR #2**	**256**
**Intervention and Comparator (IC)**		
#4	"risk score"	3,009
#5	"risk prediction"	7,544
#6	"risk model"	37,629
#7	questionnaire	16,475
#8	#4 OR #5 OR #6 OR #7	57,291
#9	predicting	202,868
#10	"prediction tool"	11,831
#11	"prediction rule"	4,948
#12	"predictive model"	43,224
#13	"prediction model"	115,789
#14	#9 OR #10 OR #11 OR #12 OR #13	309,723
#15	"clinical decision model"	2,075
#16	"clinical decision rule"	334
#17	"clinical prediction score"	547
#18	"decision support systems"	21,922
#19	#15 OR #16 OR #17 OR #18	23,930
#20	"artificial intelligence"	42,061
#21	"machine learning"	132,880
#22	#20 OR #21	165,897
**#23**	**#8 OR #14 OR #19 OR #22**	**501,936**
**Outcome (O)**		
#24	"medication overuse"	5
#25	"medication overuse headache"	2
#26	"medication over*"	691
#27	"medication misuse"	36
#28	"MOH"	76
#29	"drug-induced headache"	0
#30	"analgesic rebound headache"	0
**#31**	**#24 OR #25 OR #26 OR #27 OR #28 OR #29 OR #30**	798
**Combine P, IC, and O**		
**#32**	**#3 AND #23 AND #31**	**5**
