# Supplementary material for: Prediction models for identifying medication overuse or medication overuse headache in migraine patients: a systematic review
Source: J Headache Pain. 2024 Oct 4;25(1):165. doi: 10.1186/s10194-024-01874-4 (PMC11450990; doi:10.1186/s10194-024-01874-4)
Supplement: Supplementary file 2 — Supplementary Material 2. [file 10194_2024_1874_MOESM2_ESM.pdf]

Appendix 2 Details of outcomes and definitions used in each study

Study	Outcome term	Definition	Criteria
Grande RB, 2009[1]	MO	A. Headache present on ≥15 days/month.<br>B. Regular overuse for >3 months of one or more acute/symptomatic treatment drugs as defined under subforms:<br>1. Ergotamine, triptans, opioids or combination analgesic medication on ≥10 days/month on a regular basis for ≥3 months.<br>2. Simple analgesics or any combination of ergotamine, triptans, analgesics or opioids on >15 days/month on a regular basis for ≥3 months without overuse of any single class alone.<br>C. Headache has developed or markedly worsened during medication overuse.	ICHD-II and relevant revisions[2-5]
Onaya T, 2013[6]	MOH	A. Headache present on ≥15 days/month.<br>B. Regular overuse for >3 months of one or more acute/symptomatic treatment drugs as defined under sub forms of 8.2.<br>1. Ergotamine, triptans, opioids, or combination analgesic medications on ≥10 days/month on a regular basis for >3 months.<br>2. Simple analgesics or any combination of ergotamine, triptans, analgesics opioids on ≥15 days/month on a regular basis for >3 months without overuse of any single class alone.<br>C. Headache has developed or markedly worsened during medication overuse.	ICHD-II with revision[4]
Mose LS, 2018[7]	MOH	A. Headache occurring on ≥15 days per month in a patient with a pre-existing headache disorder.<br>B. Regular overuse for >3 months of one or more drugs that can be taken for acute and/or symptomatic treatment of headache.<br>C. Not better accounted for by another ICHD-3 diagnosis.	ICHD-III beta[8]
Ferroni P, 2020[9]	MO	-	Not provide references
Wang YF, 2023[10]	MOH	A. Headache occurring on ≥15 days/month in a patient with a pre-existing headache disorder.<br>B. Regular overuse for >3 months of one or more drugs that can be taken for acute and/or symptomatic treatment of headache.<br>C. Not better accounted for by another ICHD-3 diagnosis.	ICHD-III[11]
Wang YF, 2023[12]	MOH	A. Headache occurring on ≥15 days/month in a patient with a pre-existing headache disorder.<br>B. Regular overuse for >3 months of one or more drugs that can be taken for acute and/or symptomatic treatment of headache.<br>C. Not better accounted for by another ICHD-3 diagnosis.	ICHD-III[11]

Abbreviations: ICHD, The International Classification of Headache Disorders; MO, medication overuse; MOH, medication overuse headache

References

1. Grande RB, Aaseth K, Saltyte Benth J, et al (2009) The Severity of Dependence Scale detects people with medication overuse: the Akershus study of chronic headache. J Neurol Neurosurg Psychiatry 80:784-789

2. (2004) The International Classification of Headache Disorders: 2nd edition. Cephalalgia 24 Suppl 1:9-160
3. Erratum (2006) Cephalalgia 26:360
4. Olesen J, Bousser M-G, Diener H-C, et al (2006) New Appendix Criteria Open for a Broader Concept of Chronic Migraine. Cephalalgia 26:742-746
5. Silberstein SD, Olesen J, Bousser MG, et al (2005) The International Classification of Headache Disorders, 2nd Edition (ICHD-II)--revision of criteria for 8.2 Medication-overuse headache. Cephalalgia 25:460-465
6. Onaya T, Ishii M, Katoh H, et al (2013) Predictive index for the onset of medication overuse headache in migraine patients. Neurol Sci 34:85-92
7. Mose LS, Pedersen SS, Debrabant B, et al (2018) The role of personality, disability and physical activity in the development of medication-overuse headache: a prospective observational study. J Headache Pain 19:39
8. (2013) The International Classification of Headache Disorders, 3rd edition (beta version). Cephalalgia 33:629-808
9. Ferroni P, Zanzotto FM, Scarpato N, et al (2020) Machine learning approach to predict medication overuse in migraine patients. Comput Struct Biotechnol J 18:1487-1496
10. Wang YF, Tzeng YS, Yu CC, et al (2023) Clinical Utility of Leeds Dependence Questionnaire in Medication-Overuse Headache. Diagnostics (Basel) 13
11. (2018) Headache Classification Committee of the International Headache Society (IHS) The International Classification of Headache Disorders, 3rd edition. Cephalalgia 38:1-211
12. Wang YF, Tzeng YS, Yu CC, et al (2023) Sex differences in the clinical manifestations related to dependence behaviors in medication-overuse headache. J Headache Pain 24:145
