# Supplementary material for: Prediction models for identifying medication overuse or medication overuse headache in migraine patients: a systematic review
Source: J Headache Pain. 2024 Oct 4;25(1):165. doi: 10.1186/s10194-024-01874-4 (PMC11450990; doi:10.1186/s10194-024-01874-4)
Supplement: Supplementary file 3 — Supplementary Material 3. [file 10194_2024_1874_MOESM3_ESM.pdf]

Appendix 3 Details of predictors considered in prediction model in each study

Predictors considered in model	Study					
	Grande RB, 2009[1]	Onaya T, 2013[2]	Mose LS, 2018[3]	Ferroni P, 2020[4]	Wang YF, 2023[5]	Wang YF, 2023[6]
**Patient demographics**						
Gender	-	C	C	C	-	-
Age	-	S	S	S	-	-
Marital status	-	-	C	-	-	-
Education	-	-	C, S	-	-	-
Occupation	-	-	C	-	-	-
Physical activity	-	-	S	-	-	-
Sleep habit	-	-	S	-	-	-
Alcohol and coffee	-	-	-	C	-	-
Smoking	-	-	-	C	-	-
Dietary intake	-	C	-	-	-	-
Menopausal status	-	-	-	C	-	-
Age at menarche	-	-	-	S	-	-
**Family history**						
Migraine in family	-	C	-	C	-	-
**Physical examinations**						
BP	-	-	-	S	-	-
BMI	-	-	-	S	-	-
**Migraine and related characteristics**						
Type of migraine	-	C	-	C	-	-
Age of onset migraine	-	C	-	S	-	-
Length of chronicization	-	-	-	S	-	-
Headache frequency	-	-	-	S	-	-
Pain localization	-	C	-	C	-	-
Characteristics of pain	-	C	-	-	-	-
Unilateral cranial autonomic symptoms	-	-	-	C	-	-
Dopaminergic symptoms	-	-	-	C	-	-
Concomittant with CH	-	-	-	C	-	-
Concomittant with TTH	-	-	C	C	-	-
Relation with menstruation	-	C	-	-	-	-
Relation with stress and uneasiness	-	C	-	-	-	-
No. of consultation with headache clinic	-	-	S	-	-	-
**Underlying diseases and symtoms**						
Depression	-	C	-	-	-	-
Neuropsychiatric	-	-	-	C	-	-
Cardiovascular	-	-	-	C	-	-
Endocrine-metabolic	-	-	-	C	-	-
Motion sickness in current	-	C	-	-	-	-
Motion sickness in childhood	-	C	-	-	-	-
**Laboratories**						
CBC panel, WBC	-	-	-	S	-	-
CBC panel, RBC	-	-	-	S	-	-
CBC panel, Neutrophils	-	-	-	S	-	-
CBC panel, Lymphocytes	-	-	-	S	-	-
CBC panel, Monocytes	-	-	-	S	-	-
CBC panel, Hct	-	-	-	S	-	-
CBC panel, Hb	-	-	-	S	-	-

CBC panel, Plt	-	-	-	S	-	-
CBC panel, MPV	-	-	-	S	-	-
Chem, INR	-	-	-	S	-	-
Chem, PT	-	-	-	S	-	-
Chem, aPTT	-	-	-	S	-	-
Chem, HbACc	-	-	-	S	-	-
Chem, Fibrinogen	-	-	-	S	-	-
Chem, FBS	-	-	-	S	-	-
Chem, Fasting insulin	-	-	-	S	-	-
Lipid panel, TC	-	-	-	S	-	-
Lipid panel, HDL	-	-	-	S	-	-
Lipid panel, LDL	-	-	-	S	-	-
Lipid panel, TG	-	-	-	S	-	-
Renal panel, Creatinine	-	-	-	S	-	-
Renal panel, BUN	-	-	-	S	-	-
LFT panel, ALT	-	-	-	S	-	-
LFT panel, AST	-	-	-	S	-	-
LFT panel, GGT	-	-	-	S	-	-
LFT panel, Total bilirubin	-	-	-	S	-	-
**Genetics**						
5-HT-TLPR	-	C	-	-	-	-
5-HT-TVNTR	-	C	-	-	-	-
5-HT2A-T102C	-	C	-	-	-	-
5-HT1B-G861C	-	C	-	-	-	-
MAOA-VNTR	-	C	-	-	-	-
MAOA-T941G	-	C	-	-	-	-
MTHFR-C677T	-	C	-	-	-	-
ACE-I/D	-	C	-	-	-	-
ESR1-G325C	-	C	-	-	-	-
ESR1-G594A	-	C	-	-	-	-
DRD2-C939T	-	C	-	-	-	-
DBH-19-bp I/D	-	-	-	C	-	-
BDNF-G196A	-	C	-	-	-	-
**Medications**						
Class of acute medication (NSAIDs, triptans, or others)	-	-	-	C	-	-
Response to triptans	-	-	-	C	-	-
Use of preventive medication	-	-	-	C	-	-
COCs	-	-	-	C	-	-
**Questionnaire-based evaluations**						
**Migraine disability**						
**Migraine Disability Assessment Score (MIDAS)**						
MIDAS-score	-	-	S	-	-	-
MIDAS-frequency	-	-	S	-	-	-
MIDAS-intensity	-	-	S	-	-	-
**Dependence behavior**						
**Modified Severity of Dependence Scale (SDS)**						
Do you think your use of your headache medication was out of control?	S	-	-	-	-	S
Did the prospect of missing a dose make you anxious or worried?	S	-	-	-	-	S
Did you worry about your use of your headache medication?	S	-	-	-	-	S

Did you wish you could stop?	S					S
How difficult did you find it to stop or go without your headache medication?	S	-	-	-	-	S
**Modified Leeds Dependence Questionnaire (LDQ)**						
Do you find yourself thinking about when you will next be able to take analgesics?	-	-	-	-	S	S
Is taking analgesics more important than anything else you might do during the day?	-	-	-	-	S	S
Do you feel your need for analgesics is too strong to control?	-	-	-	-	S	S
Do you plan your days around taking analgesics?	-	-	-	-	S	S
Do you take analgesic in a particular way in order to increase the effect it gives you?	-	-	-	-	S	S
Do you take analgesics morning, afternoon and evening?	-	-	-	-	S	S
Do you feel you have to carry on taking analgesics once you have started?	-	-	-	-	S	S
Is getting the effect you want more important than the particular analgesic you use?	-	-	-	-	S	S
Do you want to take more analgesics when the effect starts to wear off?	-	-	-	-	S	S
Do you find it difficult to cope with life without analgesics?	-	-	-	-	S	S
**Personality trait**						
**NEO Five-Factor Inventory (NEO-FFI) personality questionnaire**						
Neuroticism	-	S	S	-	-	-
Extraversion	-	S	S	-	-	-
Openness	-	S	S	-	-	-
Agreeableness	-	S	S	-	-	-
Conscientiousness	-	S	S	-	-	-

Abbreviations: ALT, alanine aminotransferase; aPTT, activated partial thromboplastin time; AST, aspartate aminotransferase; BMI, body mass index; BP, blood pressure; BUN, blood urea nitrogen; CBC, complete blood count; CH, cluster headache; Chem, blood chemistry; COCs, combined oral contraceptives; FBS, fasting blood sugar; GGT, gamma-glutamyl transferase; Hb, hemoglobin; HbA1c, hemoglobin A1C; Hct, hematocrit; HDL, high-density lipoprotein; INR, International Normalized Ratio; LDL, low-density lipoprotein; LFT, liver function test; MPV, mean platelet volume; Plt, platelet; PT, prothrombin time; RBC, red blood cell; TC, total cholesterol; TG, trigleceride; TTH, tension-type headache; WBC, white blood cell; C, used in categorized form; S, used in scaled form

References

1. Grande RB, Aaseth K, Saltyte Benth J, et al (2009) The Severity of Dependence Scale detects people with medication overuse: the Akershus study of chronic headache. J Neurol Neurosurg Psychiatry 80:784-789
2. Onaya T, Ishii M, Katoh H, et al (2013) Predictive index for the onset of medication overuse headache in migraine patients. Neurol Sci 34:85-92
3. Mose LS, Pedersen SS, Debrabant B, et al (2018) The role of personality, disability and physical activity in the development of medication-overuse headache: a prospective observational study. J Headache Pain 19:39
4. Ferroni P, Zanzotto FM, Scarpato N, et al (2020) Machine learning approach to predict medication overuse in migraine patients. Comput Struct Biotechnol J 18:1487-1496
5. Wang YF, Tzeng YS, Yu CC, et al (2023) Clinical Utility of Leeds Dependence Questionnaire in Medication-Overuse Headache. Diagnostics (Basel) 13
6. Wang YF, Tzeng YS, Yu CC, et al (2023) Sex differences in the clinical manifestations related to dependence behaviors in medication-overuse headache. J Headache Pain 24:145
